# Biofilm formation of the black yeast-like fungus *Exophiala dermatitidis* and its susceptibility to antiinfective agents

**DOI:** 10.1038/srep42886

**Published:** 2017-02-17

**Authors:** Lisa Kirchhoff, Maike Olsowski, Katrin Zilmans, Silke Dittmer, Gerhard Haase, Ludwig Sedlacek, Eike Steinmann, Jan Buer, Peter-Michael Rath, Joerg Steinmann

**Affiliations:** 1 Institute of Medical Microbiology, University Hospital Essen, University of Duisburg-Essen, Essen, Germany; 2 Institute of Medical Microbiology, Rheinisch-Westfälische Technische Hochschule Aachen University Hospital, Aachen, Germany; 3 Institute of Medical Microbiology and Hospital Epidemiology, Medical School Hannover (MHH), Hannover, Germany; 4 Institute for Experimental Virology, TWINCORE Centre for Experimental and Clinical Infection Research; a joint venture between the Medical School Hannover (MHH) and the Helmholtz Centre for Infection Research (HZI), Hannover, Germany.

## Abstract

Various fungi have the ability to colonize surfaces and to form biofilms. Fungal biofilm-associated infections are frequently refractory to targeted treatment because of resistance to antifungal drugs. One fungus that frequently colonises the respiratory tract of cystic fibrosis (CF) patients is the opportunistic black yeast–like fungus *Exophiala dermatitidis*. We investigated the biofilm-forming ability of *E. dermatitidis* and its susceptibility to various antiinfective agents and natural compounds. We tested 58 *E. dermatitidis* isolates with a biofilm assay based on crystal violet staining. In addition, we used three isolates to examine the antibiofilm activity of voriconazole, micafungin, colistin, farnesol, and the plant derivatives 1,2,3,4,6-penta-O-galloyl-b-D-glucopyranose (PGG) and epigallocatechin-3-gallate (EGCG) with an XTT reduction assay. We analysed the effect of the agents on cell to surface adhesion, biofilm formation, and the mature biofilm. The biofilms were also investigated by confocal laser scan microscopy. We found that *E. dermatitidis* builds biofilm in a strain-specific manner. Invasive *E. dermatitidis* isolates form most biomass in biofilm. The antiinfective agents and the natural compounds exhibited poor antibiofilm activity. The greatest impact of the compounds was detected when they were added prior cell adhesion. These findings suggest that prevention may be more effective than treatment of biofilm-associated *E. dermatitidis* infections.

The fungus *Exophiala dermatitidis* frequently colonises the respiratory tract of cystic fibrosis (CF) patients. Numerous studies have reported that the rate of occurrence of *E. dermatitidis* in CF patients ranges from 1% to 19%^1^,^2^. In addition, *E. dermatitidis* causes phaeohyphomycosis in immunosuppressed patients and in the central nervous system of immunocompetent Asian patients^3^,^4^. Outside the human body, *E. dermatitidis* occurs in warm and humid areas and is therefore believed to originate in tropical climates^5^. It is also encountered worldwide in the man-made environment, for example in dishwashers, steam baths and sauna facilities^6^.

*Exophiala dermatitidis* is metabolically active over a wide range of temperatures and is also known to be stable at extreme pH values^7^. Belonging to the family of black yeast-like fungi, *E. dermatitidis* is characterized by a melanized thick multi-layered cell wall. This darkly pigmented cell wall is linked with resistance to antifungal agents and extreme environmental conditions^8^. In addition, its dimorphic character is associated with pathogenicity^9^. The ability of this yeast to switch morphologically from the yeast state to the hyphae state is a virulence factor and an indicator of biofilm formation, because this switch is part of the biofilm formation process, as showed for *Candida* spp.^10^.

The life form “biofilm” prohibits the clearance of infections and results in chronic recurrent infections^11^. The embedded life mode of the microbes in the extracellular matrix of the biofilm protects the fungus against the host defence and antiinfective agents. A recent study found that *E. dermatitidis* can form biofilm. Sav *et al*., in an investigation of the biofilm behavior of 137 environmental and 7 clinical *E. dermatitidis* isolates, detected the ability of *E. dermatitidis* to form biofilm in 15% of environmental isolates and 29% of clinical isolates^12^.

We analyzed a set of 58 *E. dermatitidis* strains of various origins (CF, environmental, invasive) and compared their ability to form biofilm. Furthermore, we tested the antibiofilm activity of several antiinfective agents, the quorum-sensing molecule (QSM) farnesol, and two natural compounds with antibiofilm activity.

## Results

### *Exophiala dermatitidis* is a biofilm builder

**Figure 1:**
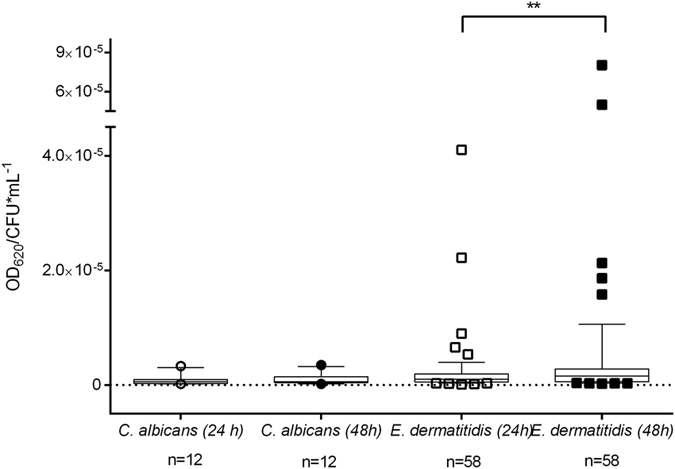
Relative biofilm formation of *Candida albicans* (ATCC 90028) and various isolates of *Exophiala dermatitidis*. Box & whiskers with 10–90 percentile. The biofilm formation wasdetected by staining with crystal violet (0.1%) for 20 minutes. Biofilm formation at 35 °C over 24 and 48 hours. Each data point represents the mean of at least three independent experiments. *P < 0.05 as determined by Student’s *t*-test. OD_620_ = optical density at 620 nm. CFU = colony-forming units.

**Figure 2:**
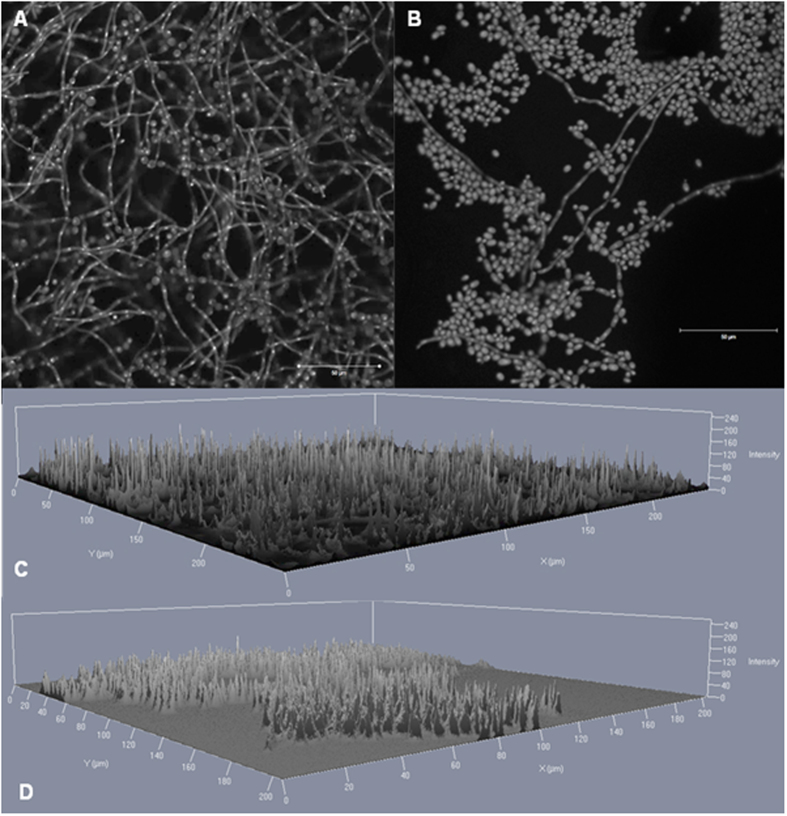
Confocal laser scan microscopy images of *Exophiala dermatitidis* biofilm grown for 48 hours at 35 °C. The DNA of the cells was stained by 0.01% acridine orange for 2 minutes. (**A**) Matured biofilm of isolate P2 (CBS 116372) in a 2D image. (**B**) Matured biofilm of isolate CF2 (CBS 552.90) in a 2D image. (**C**) Matured biofilm of isolate P2 in a 2.5 D image. (**D**) Matured biofilm of isolate CF2 in a 2.5D image. Scale bar equals 50 μm. A laser with a wavelength of 488 nm was used.

**Figure 3:**
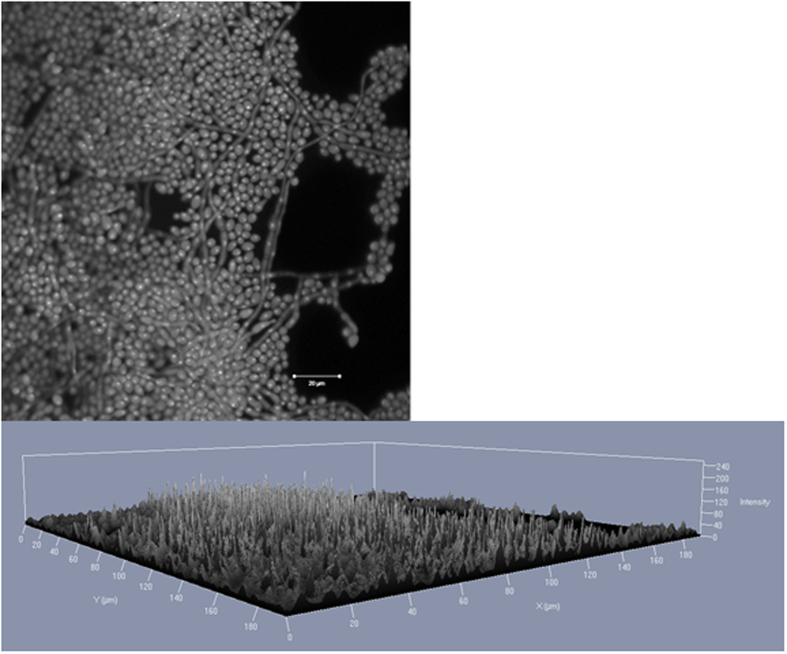
Confocal laser scan microscopy images of *C. albicans* (ATCC 90028) biofilm grown for 48 hours at 35 °C. The DNA of the cells was stained by 0.01% acridine orange for 2 minutes. A laser with a wavelength of 488 nm was used. (**A**) Matured biofilm in a 2D image. Scale bar equals 50 μm. (**B**) Matured biofilm a 2.5 D image.

To evaluate the biofilm formation capability of *E. dermatitidis,* we performed a biofilm formation assay using a total of 58 isolates of various origins. Of these isolates, 15 originated from non-CF patients, 35 originated from CF patients, and 8 originated from the environment. The crystal violet (CV)-based assay showed that *E. dermatitidis* was able to form biofilm. Of note, biofilm formation turned out to be strain specific, with higher amount of biomass in biofilm after 48 hours than after 24 hours (*P* = 0.0098). A total of 36% of the tested *E. dermatitidis* isolates formed biofilms with a biomass equal to or higher than the biomass of the biofilms formed by *C. albicans* (Figs 1, 2 and 3).

**Figure 4:**
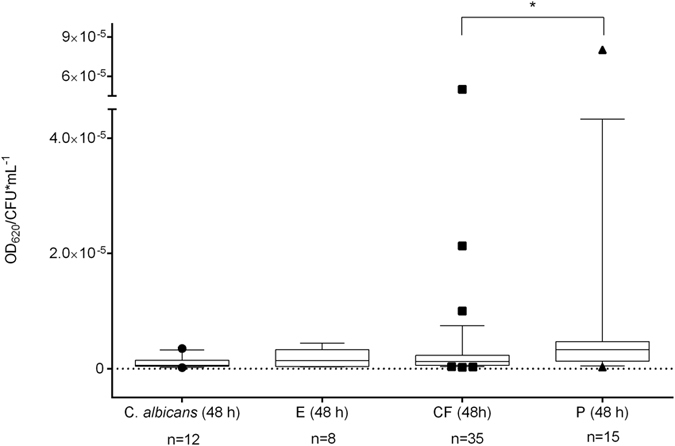
Relative biofilm formation of *Candida albicans* (ATCC 90028) and various isolates of *Exophiala dermatitidis.* Box & whiskers with 10–90 percentile. E = environmental origin. CF = isolate from cystic fibrosis (CF) patient. P = invasive isolate from non-CF patient. Relative biofilm formation detected by staining with crystal violet (0.1%) for 20 minutes after biofilm formation at 35 °C over 48 hours. Each data point represents the mean of at least three independent experiments. *P < 0.05 as determined by Student’s *t*-test. CFU = colony-forming units.

The invasive isolates from non-CF patients exhibited significantly more biomass in biofilm compared to isolates from CF patients (*P* = 0.0256). The biomass amount of invasive isolates tends to be higher than the biomass involved in biofilm of isolates from other origins (Fig. 4) This was also detected in the confocal laser scan microscopy (CLSM) (Fig. 2). The biofilm of the invasive isolate P2 contained more hyphal structures and was more clustered than the biofilm of the CF-patients isolate (CF2). The 2.5 D images from the invasive isolate, created in the CLSM, displayed higher signal intensity when compared to the CF-patients isolate (Fig. 2C,D).

### XTT reduction assay is suitable for metabolically active biofilm detection

Because the CV-based biofilm formation assay can detect only the biomass in biofilm, a 2, 3-bis (2-methoxy-4-nitro-5-sulfophenyl)-5[(phenylamino) carbonyl] – 2Htetrazolium hydroxide (XTT) reduction assay was introduced to investigate the metabolic activity of the biofilm. This assay was carried out for studying antibiofilm activity. Therefore, we investigated the suitability of the XTT assay for detecting the metabolic activity of *E. dermatitidis* biofilm. Metabolic activity of biofilm formation was measurable over 48 hours. Heat-inactivated cells showed no significant results upon XTT reduction.

**Figure 5:**
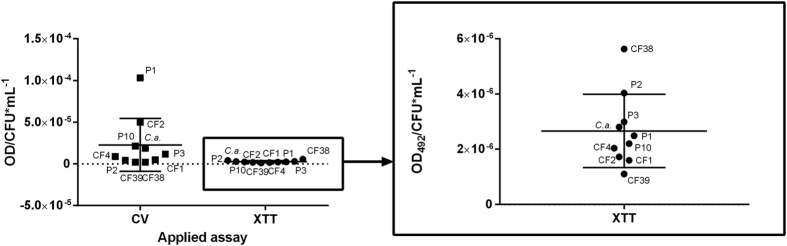
*Exophiala dermatitidis* biofilm formation. Optical density at 620 nm (OD_620_) as measured after staining with crystal violet (CV), and optical density at 492 nm (OD_492_) after XTT reduction. Each data point represents the mean of at least three independent experiments. P1, P2, P10 = invasive *E. dermatitidis* isolates from non-CF patients. CF1, CF2, CF4, CF39, CF38 = *E. dermatitidis* isolates from CF patients. *C.a.* = *Candida albicans.* CFU = colony-forming units.

To understand the correlation, if any, between the metabolic activity and the biomass of *E. dermatitidis* biofilm, we compared the results of the CV assay and the XTT assay. The comparison of metabolic activity as detected by the XTT assay and of biomass, detected by CV staining, showed that the metabolic activity of *E. dermatitidis* biofilm is not linearly associated with the biomass involved in biofilm. Thus, the two assays are complementary procedures (Fig. 5).

### *Exophiala dermatitidis* biofilms are mostly resistant against antibiofilm agents

Biofilm formation is a survival strategy by which fungi adapt to their environment. Thus, treating an infection caused by a biofilm-forming organism is difficult. To detect substances that may inhibit *E. dermatitidis* biofilm, we tested the effect of six possible antibiofilm agents on *E. dermatitidis* cell-adhesion, biofilm formation, and mature biofilm with the introduced XTT assay. We also tested the antibiofilm activity of these six compounds on *C. albicans* biofilm as a control. Before doing so, we determined the minimum inhibitory concentrations (MICs) of the substances and respectively the minimum eradication concentration (MEC) for micafungin by broth microdilution method to identify the range for antibiofilm activity testing (Table 1).

In general, *E. dermatitidis* biofilm exhibited a higher resistance to the tested compounds than did *C. albicans.* Compared to the addition of agents on cells that were already adherent, the antibiofilm activity of all tested agents was higher before cell-surface adherence and when they were added to mature biofilm. The process of *E. dermatitidis* biofilm formation, which usually occurs 2 to 48 hours after inoculation, showed higher resistance against treatment with antiinfective agents and natural substances.

**Figure 6:**
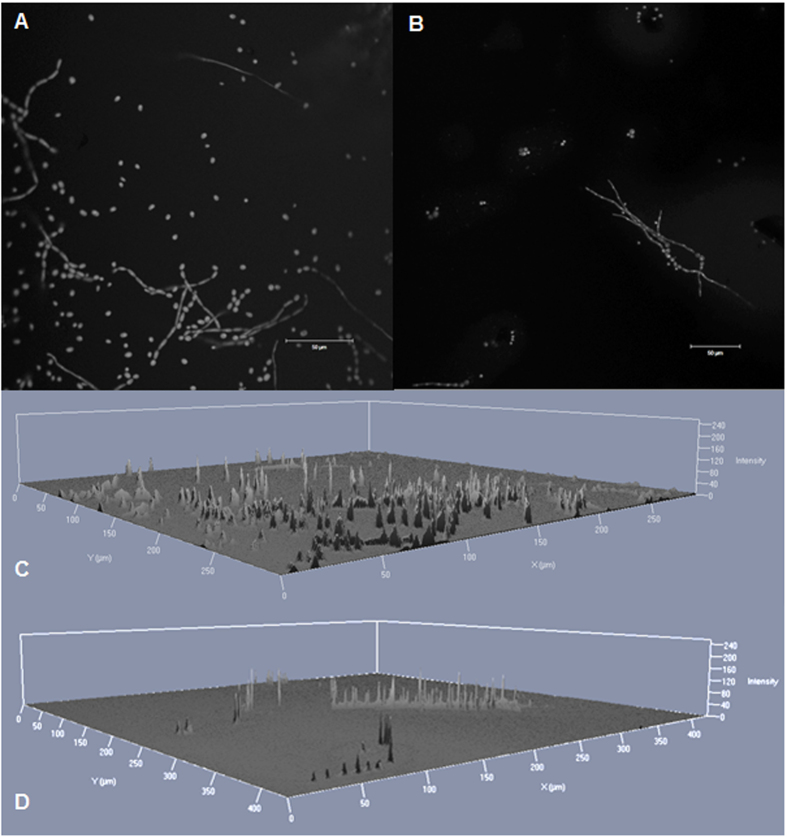
Confocal laser scan microscopy images of *E. dermatitidis* isolate P2 (CBS 116372) biofilm, formed in the presence of antiinfective agents. Biofilm was grown for 48 hours at 35 °C in the presence of (**A**,**C**) 64 mg/L colistin, (**B**,**D**) 8 mg/L micafungin. 2D (**A**,**B**) and 2.5 D (**C**,**D**) images were taken. The DNA of the cells was stained by 0.01% acridine orange for 2 minutes. Scale bar equals 50 μm. A laser with a wavelength of 488 nm was used.

**Figure 7:**
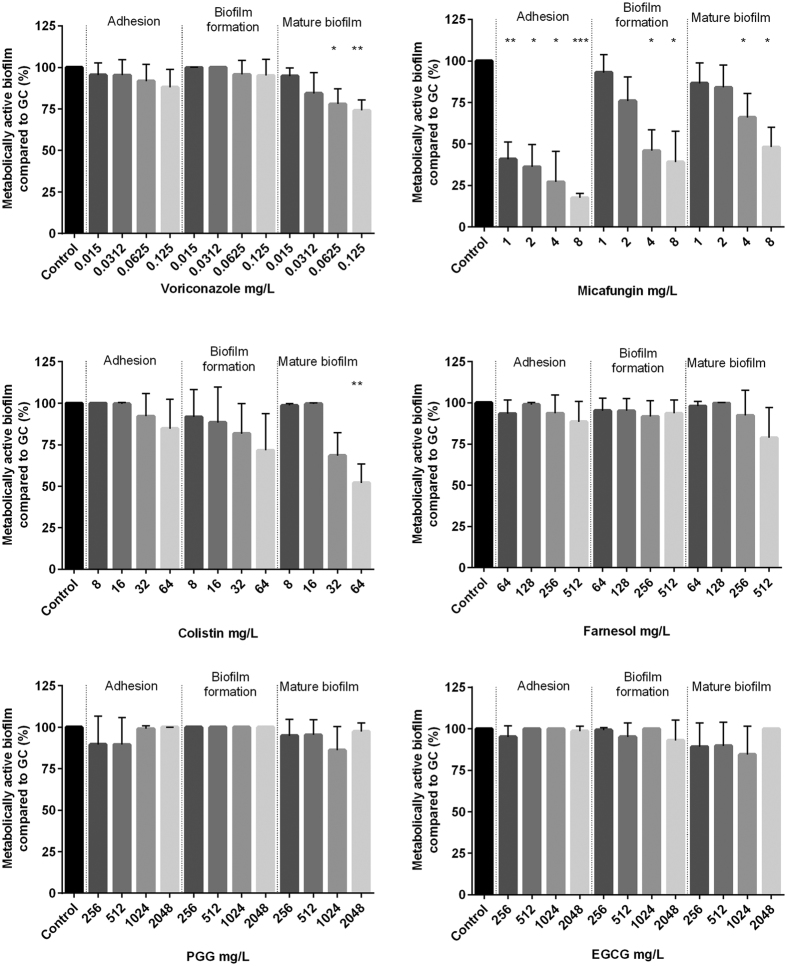
Growth (mean with standard deviation in %) of *E. dermatitidis* (P1) biofilm after treatment with voriconazole (**A**), micafungin (**B**), colistin (**C**), farnesol (**D**), epigallocatechin gallate (EGCG, **E**) and 1,2,3,4,6-penta-O-galloyl-β-d-glucose (PGG, **F**) in the concentrations indicated on the *x*-axis. Control = growth control without treatment. Adhesion = addition of drug at time point 0. Biofilm formation = drug addition after 2 hours. Mature biofilm = drug addition after 48 hours of biofilm formation. Growth was evaluated by XTT assay; optical density readings at 492 nm (OD_492_) were measured. *P < 0.05; **P < 0.05; ***P < 0.001. n = 4.

**Figure 8:**
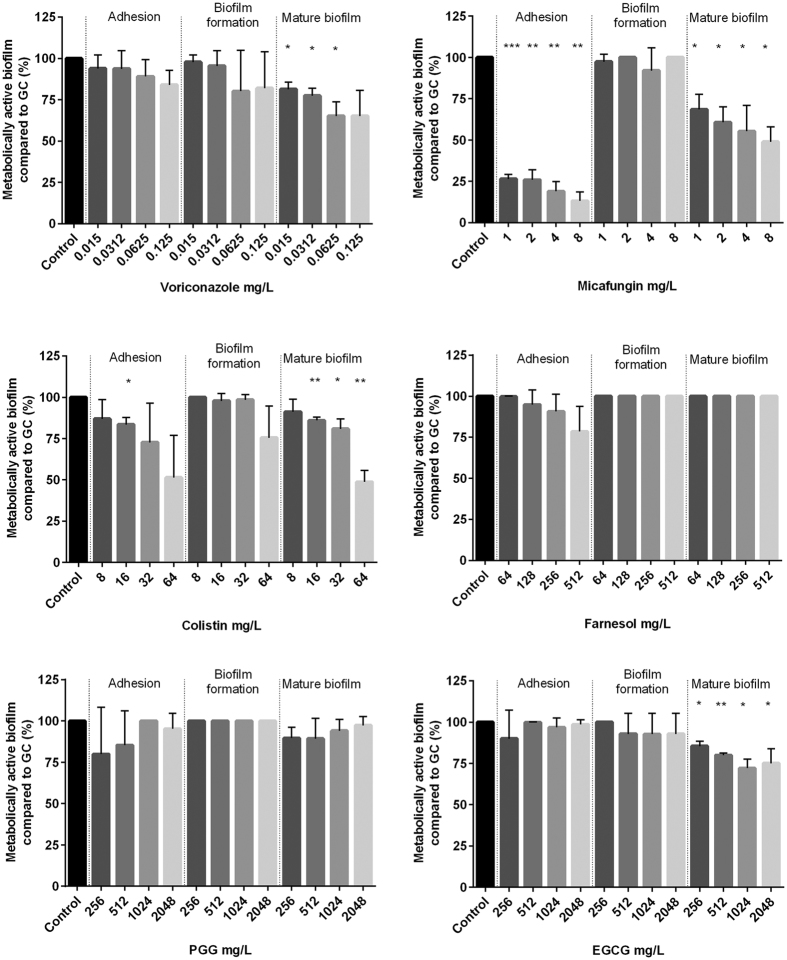
Growth (mean with standard deviation in %) of *E. dermatitidis* (P2) biofilm after treatment with voriconazole (**A**), micafungin (**B**), colistin (**C**), farnesol (**D**), epigallocatechin gallate (EGCG, **E**) and 1,2,3,4,6-penta-O-galloyl-β-d-glucose (PGG, **F**) in the concentrations indicated on the *x*-axis. Control = growth control without treatment. Adhesion = addition of drug at time point 0. Biofilm formation = drug addition after 2 hours. Mature biofilm = drug addition after 48 hours of biofilm formation. Growth was evaluated by XTT assay; optical density readings at 492 nm (OD_492_) were measured. *P < 0.05; **P < 0.05; ***P < 0.001. n = 4.

**Figure 9:**
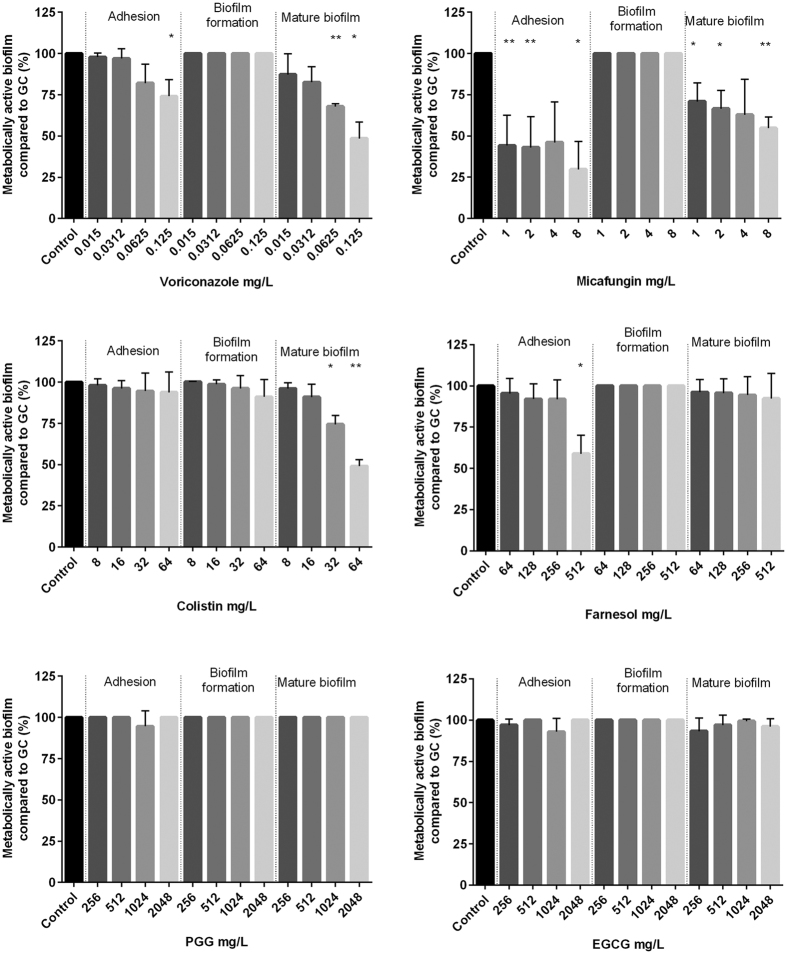
Growth (mean with standard deviation in %) of *E. dermatitidis* (CF2) biofilm after treatment with voriconazole (**A**), micafungin (**B**), colistin (**C**), farnesol (**D**), epigallocatechin gallate (EGCG, **E**) and 1,2,3,4,6-penta-O-galloyl-β-d-glucose (PGG, **F**) in the concentrations indicated on the *x*-axis. Control = growth control without treatment. Adhesion = addition of drug at time point 0. Biofilm formation = drug addition after 2 hours. Mature biofilm = drug addition after 48 hours of biofilm formation. Growth was evaluated by XTT assay; optical density readings at 492 nm (OD_492_) were measured. *P < 0.05; **P < 0.05; ***P < 0.001. n = 4.

**Figure 10:**
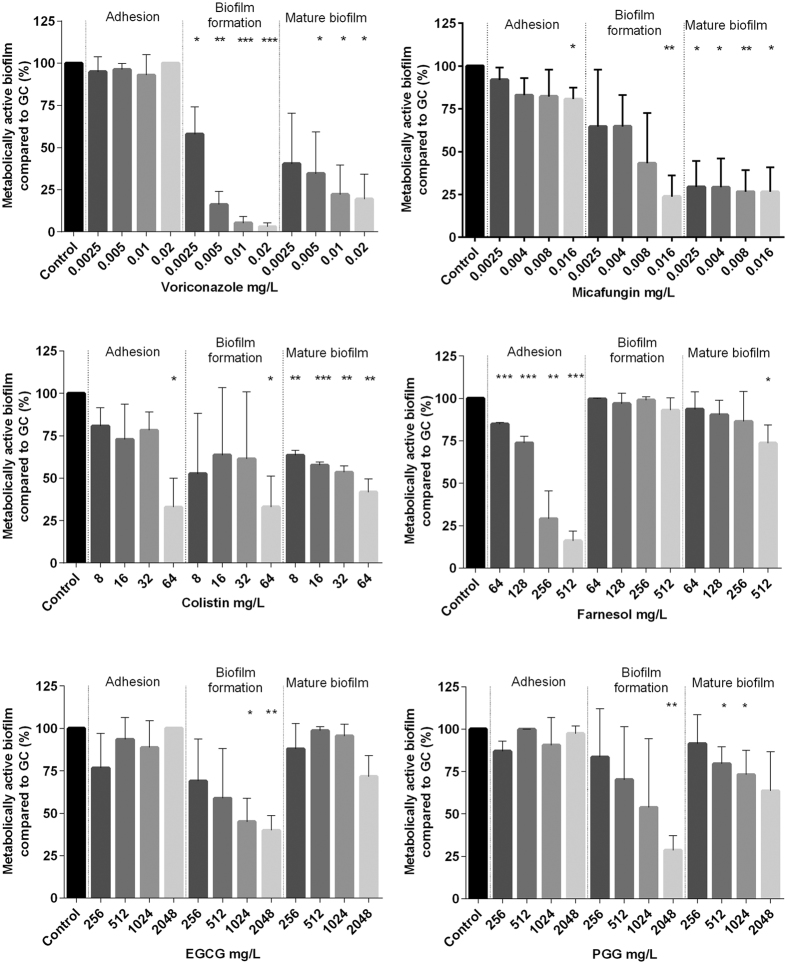
Growth (mean with standard deviation in %) of *C. albicans* (ATCC 90028) biofilm after treatment with voriconazole (**A**), micafungin (**B**), colistin (**C**), farnesol (**D**), epigallocatechin gallate (EGCG, **E**) and 1,2,3,4,6-penta-O-galloyl-β-d-glucose (PGG, **F**) in the concentrations indicated on the *x*-axis. Control = growth control without treatment. Adhesion = addition of drug at time point 0. Biofilm formation = drug addition after 2 hours. Mature biofilm = drug addition after 48 hours of biofilm formation. Growth was evaluated by XTT assay; optical density readings at 492 nm (OD_492_) were measured. *P < 0.05; **P < 0.05; ***P < 0.001. n = 3.

The best antibiofilm activity against *E. dermatitidis* was exhibited by the antifungal agent micafungin. The second highest growth reduction resulted from treatment with the antibiotic colistin. The antibiofilm effect of both agents was also documented in the CLSM (Fig. 6). An additional analysis correlates with the results of this assay. The biofilm was reduced by approximately 90% when treated with micafungin and 62% when treated with colistin as detected by gray-value measurements of the CLSM created images. In contrast, the natural substances PGG and EGCG had no visible effect on biofilm formation of *E. dermatitidis* at any step in the process (Figs 7, 8 and 9). On the other hand, treatment with PGG and EGCG affected the control organism *C. albicans* in the process of biofilm development (Fig. 10). At a concentration of 512 mg/L, the QSM farnesol decreased adhesion by approximately 25% in average. In contrast, we detected no significant difference between the non-treated growth control and either the cells treated during biofilm formation or the preformed biofilm (Figs 7, 8 and 9). However, farnesol inhibits the cell adhesion of *C. albicans* and exerts an antibiofilm effect on 48-hour preformed *C. albicans* biofilm (Fig. 10).

The antifungal agent voriconazole reduced the growth of *E. dermatitidis* biofilm during adhesion and also reduced the growth of mature biofilm. The decrease in the number of viable cells was visible for all three strains treated with voriconazole. However, when different concentrations of voriconazole were applied to the different strains, the growth of mature biofilm was reduced at different rates. Thus, the activity of voriconazole was also strain specific (Supplementary Fig. S1). The antibiofilm activity of voriconazole was higher against the CF isolate than against the invasive isolates.

### The antibiofilm activity of all agents is strain specific

Overall, strain-specific MICs/MEC of *E. dermatitidis* were investigated for both planktonic and biofilm cells (Supplementary Fig. S1). Treatment with 0.06 mg/L voriconazole inhibited mature biofilm by 37%. In contrast, the planktonic *E. dermatitidis* isolates P2 and CF2 exhibited a high MIC (>16 mg/L) against voriconazole. Voriconazole had a MIC of 0.25 mg/L against planktonic P1 cells, as detected by the microdilution method (Table 1). When the strain-specific susceptibility of biofilm was analysed, the isolate P1 exhibited the lowest biofilm reduction rate. After treatment with 0.06125 mg/L voriconazole, the biofilm of this isolate was at 78% of the growth controls. P2 (65%) and CF2 (68%) were also reduced in comparison with the growth controls. Therefore, the planktonic susceptibility and the biofilm susceptibility are not directly dependent on each other within one strain.

### Combination of colistin and micafungin showed indifferent effects on E. dermatitidis biofilm

Synergistic operating agents offer the opportunity to decrease the necessary drug uptake, thus also reducing adverse effects and minimizing the risk of emerging resistance. Micafungin and colistin exhibited the highest antibiofilm activity against *E. dermatitidis* (Figs 7, 8 and 9). Therefore, combination treatment with micafungin and colistin was applied in the assays, in addition to single treatment. The aim was to detect a possible synergy between the two substances. The concentrations of the two drugs were the same in combination treatment as in single treatment. The checkerboard method showed that micafungin and colistin are indifferent in treatment against *E. dermatitidis* biofilm when applied before adhesion or when applied to mature biofilm (Supplementary Tables S1 and S2).

## Discussion

In the study reported here, we systemically investigated the biofilm formation of the black yeast-like fungus *E. dermatitidis*. The CV assay showed that all tested *E. dermatitidis* isolates could form biofilm under the described conditions with a significantly higher biomass in biofilm after 48 hours of incubation. Invasive isolates produced significantly more biomass than did the isolates from CF patients. Sav *et al*. recently reported that only 15% of 137 environmental isolates and two of the seven (29%) clinical *E. dermatitidis* isolates formed biofilm over a 24-h incubation period^12^. When the evaluation method introduced by Sav *et al*.^12^ is applied to the results of this study, 86% of the total tested isolates, 63% of the environmental isolates, and 92% of the clinical isolates exhibited biofilm formation. In both studies, the clinical isolates showed a higher percentage of biofilm builders. However, the results vary widely; these findings may be due to differences in the period of biofilm formation and the growing conditions. In addition to the findings of Sav *et al*., two other publications reported biofilm capabilities of *E. dermatitidis*. However, they were limited by the number of included strains^13^ or by analyzing only isolates from non-human sources^14^.

The XTT assay and CV staining are complementary, because CV staining detects biomass in biofilm and the XTT assay detects metabolic activity^15^. The biofilm-detecting methods based on CV and XTT achieve different results, and both the quantities and the relation differ. We compared both procedures and found a 50% deviation in the results for *C. albicans*, a finding comparable to the results of a previous study. Marcos-Zambrano *et al*. tested both assays on *Candida* and non-*Candida* spp. Biofilms. They found that the overall agreement of both methods was 43.7% and that the agreement for *C. albicans* in particular was higher than 50%^15^. In our study, the difference in the quotient of optical density (OD) and colony forming units (CFUs) per mL of *E. dermatitidis* in both assays showed a high variance between biomass and metabolic activity.

*Exophiala dermatitidis* was previously identified as an exopolysaccharide producer^16^. A thick extracellular matrix can reduce the diffusion of oxygen and nutrients and thus can reduce metabolic activity^17^. A difference in the biofilm matrix structure can therefore explain the lower rates of XTT reduction^15^. In addition, because *E. dermatitidis* is a black yeast-like fungus, the thick cell wall containing melanin could influence the metabolic rate. The composition and thickness of the cell wall vary between the tested isolates, causing differences in metabolic activity. Another explanation for the weak association between biofilm mass and metabolic activity could be that some of the tested samples had already reached their maximum biomass, limiting the growth rate and the metabolic activity^17^.

An interstrain comparison with XTT is impossible. Various fungal species and various strains reduce XTT differently^18^. However, the XTT assay can measure the inhibitory effects of possible antifungal agents against *E. dermatitidis* biofilm.

The broth microdilution tests and the antibiofilm activity tests of the agents found an isolate-specific MIC/MEC as well as an isolate-specific minimum biofilm eradication concentration (MBEC). Determination of the MBECs of the agents against the various biofilm development stages showed that the treatment of *E. dermatitidis* biofilm is most effective when carried out preventive and when administered to preformed biofilms. These findings were confirmed by the study of Ramage *et al*. about the effect of the QSM farnesol on *C. albicans* biofilm formation^19^.

Biofilm formation processes are regulated by the secretion of QSMs. In the biofilm builder *C. albicans*, three QSMs have been identified, of which the most popular is farnesol. However, until now nothing was known about the existence of QSMs in the biofilm processes of *E. dermatitidis*. Farnesol was previously found to influence the processes of adhesions and the induction of biofilm dispersal of *Candida* spp.^19^. Here we found that farnesol had no antibiofilm activity against mature *E. dermatitidis* biofilm. Furthermore, the adhesion of *E. dermatitidis* cells was decreased by 25% when they were treated with 512 mg/L farnesol. The control *C. albicans* exhibited the expected reduction in biofilm growth when treated with farnesol^19^.

The antibiofilm activity of the analysed antifungal agents voriconazole and micafungin was, as expected, higher than that of the natural substances. Echinocandins have been shown to be effective against *Candida* biofilms. Micafungin exerted better antibiofilm activity against *E. dermatitidis* than did voriconazole. This finding was confirmed by the results of a study of *Candida* spp. biofilm^20^.

Colistin, a member of the polymyxin family, is an antibiotic used to treat infections involving gram-negative bacteria, targeting the bacterial membrane. The fungal cell wall and the cytoplasmic membrane serve as barriers against several agents. The *E. dermatitidis* isolates analysed in the microdilution for susceptibility testing exhibited isolate-specific MICs against colistin. With the lowest determined MIC of 64 mg/L, the *in vitro* activity of colistin was lower than expected. Previous reports stated that colistin exhibited a MIC_50_ of 12 mg/L and a MIC_90_ of 24 mg/L against *E. dermatitidis*^21^. Here, the antibiofilm effect of colistin against *E. dermatitidis* showed that colistin exerted a significant effect on cell adhesion, biofilm formation, and preformed biofilm. Schwartz *et al*. found that polymyxin affects the cell wall of fungi at high concentrations. At low concentrations, it increases the membrane permeability of fungi, enabling antifungal agents to more easily gain access to their site of action^22^.

The agents colistin and micafungin exhibited the most promising antibiofilm activity against *E. dermatitidis.* This was detected in the XTT assay as well as in the microscopy. However, combined treatment of micafungin and colistin exerted no synergistic effect against *E. dermatitidis* biofilm. In contrast, it has been shown in a previous study that the antibiotic colistin acts synergistically with antifungal agents of the echinocandin family against planktonic *Candida* species^23^. Echinocandin-mediated weakening of the cell wall may enable colistin to target the cell wall and thus reinforces the antifungal activity of echinocandins^23^. However, the combination of colistin and micafungin showed indifferent effects against *E. dermatitidis* cells during biofilm formation.

The plant derivatives EGCG and PGG were expected to exert antibiofilm activity against *E. dermatitidis*. Among other derivatives, polyphenols of green tea, especially EGCG, have been shown to exert anticarcinogenic, chemopreventive, antiatherogenic, antioxidant, and antimicrobial activity *in vitro* and *in vivo*^24^,^25^,^26^,^27^,^28^,^29^. Antifungal activity of EGCG has previously been demonstrated against planktonic *C. albicans* and against *Candida* spp. biofilm, especially when combined with antifungal agents^28^,^29^. PGG, a derivate of the *Paeonia lactiflora* root, also exerts a growth-inhibitory effect against several bacteria^30^,^31^. However, none of the natural substances, EGCG or PGG, exerted an effect on *E. dermatitidis* biofilm and neither planktonic growth of *E. dermatitidis* nor biofilm development was affected by the addition of EGCG or PGG.

Explanations for the resistance of *E. dermatitidis* against PGG and EGCG can be found in its thick and melanized cell wall at all life cycle stages. It has been previously reported that melanin enhances the resistance of several fungi^32^,^33^. In addition, experiments on the effect of polyphenols on the cell membrane and the cell wall of *C. albicans* showed that the catechins either permeabilize the cell membrane or disrupt parts of the cell wall, allowing the cells to take up substrates^28^. Because the cell wall of *E. dermatitidis* is thick and highly melanized, it is supposed to be more resistant to the effect of EGCG and PGG.

Resistance measurements of biofilm cells against all of the tested reagents at the concentrations of the detected MICs/MEC during biofilm formation demonstrated that planktonic cells are more susceptible to treatment with antiinfective agents than are cells embedded in a biofilm community. Cells embedded in the extracellular matrix of a biofilm are more likely to be resistant to host defence and antiinfective agents. Ning *et al*. studied the synergism of EGCG and antifungal agents against *Candida* spp. and found that biofilm cells exhibited significantly higher MICs (20 to 3200 times higher) than planktonic cells^29^.

In summary, we found that *E. dermatitidis* can form biofilm and that invasive isolates exhibit significantly higher biofilm-forming ability than do isolates from CF patients. The metabolic activity and the biomass involved in biofilm are strain specific.

*E. dermatitidis* biofilm is susceptible to the antifungal agents’ micafungin and voriconazole during adhesion and as a premature biofilm. The antibiotic colistin also reduced as well the fungal growth rate, especially during treatment of mature biofilms. In contrast, the natural substances EGCG and PGG had no significant effect on *E. dermatitidis* biofilm. Farnesol affects only fungal growth. Preventive treatment of the surface before biofilm formation is the most promising strategy.

## Material and Methods

### Strains

All clinical sputum samples were used after performing a conventional microbiological diagnosis. The study did not include patient’s details and did not result in additional constraints for the patients. All data were anonymously analysed without patients consent due to the retrospective nature of the study. All procedures and methods were carried out in accordance with approved guidelines.

We analysed 58 *E. dermatitidis* isolates: eight isolates (14%) from the environment, 15 (26%) invasive strains isolated from Asian patients, and 35 strains (60%) from the sputum of CF patients. As a control, we used the biofilm builder *Candida albicans* (American type collection (ATCC) 90028). To validate the XTT cell proliferation assay, we used the melanin-deficient mutant Mel^-^3 (ATCC 44504) and its corresponding wild type (ATCC 34100). For susceptibility testing, we used *E. dermatitidis* reference strains Centraalbureau voor Schimmelcultures (CBS) 109154, CBS 116372, and CBS 552.90. The species had been previously identified by sequence analyses of the internally transcribed spacer 1 (ITS1)^34^. All strains part of this study are listed in Table 2.

### Biofilm formation assay

The biofilm-forming ability of *E. dermatitidis* isolates was analysed using a CV assay. We grew 58 *E. dermatitidis* isolates and *C. albicans* over 48 hours in Sabouraud bouillon containing 2% glucose at 35 °C and high shaking conditions (200 rpm), ensuring culturing of yeast-formed cells. Cells were washed with phosphate-buffered saline (PBS). A suspension with a cell density of 1 × 10^6^ cells/mL in RPMI (pH 7.0) was set for each isolate. A 200-μL aliquot of the suspension was added to each well of a polystyrene, sterile, flat-bottomed 96-well microtiter plate. Sterile RPMI was used as a control for a blank correction.

After incubation over a period of 24 to 48 h, the plate was rinsed three times with PBS, and 125 μL of a 0.1% CV solution was added to each well. The staining was carried out for a minimum of 20 min at room temperature. Three additional rinsing steps with PBS were followed by air drying overnight. Next, 200 μL of 30% acetic acid was added to each well of the plate and incubated over 30 minutes, after which 150 μL of the solution was transferred from each well to a fresh microtiter plate. The plate was read at an OD_620_. The OD_620_ value of each well was calculated by subtraction of the blank reading. An inoculum size was estimated by CFU counting as follows: 100 μL of suspension at various dilutions was plated on malt extract agar, and the grown colonies were counted after incubation for 48 hours at 35 °C.

### Viability assay

The metabolic activity of *E. dermatitidis* biofilm was measured by using an XTT assay. Seven isolates of the eight tested exhibited a high biomass amount in biofilm whereas one exhibited a low biomass amount. Strain cultivation and biofilm formation were carried out as described above. After incubation, the plates were rinsed three times with PBS, and 100 μL of 1 μM menadione in XTT solution was added to each well. The XTT/menadione solution was prepared fresh: 0.5 mg/mL XTT (Sigma, Steinheim, Germany) in sterile NaCl (0.9%), and 10 mM menadione in ethanol (100%). The final concentration of 1 μM was achieved by the addition of 1 μL menadione stock to 10 mL XTT solution. After the addition of the XTT/menadione solution, the plates were incubated in the dark at 36 °C for 3 h, and 75 μL of solution from each well was transferred to a new, sterile, U-bottom-shaped microtiter plate for measurement of the OD_492_.

To exclude inaccuracy with regard to pigments, such as melanin deposits in the cell wall, we used the XTT assay to analyze planktonic living, heat-killed cells, and Mel^-^3 cells. We used 0.5 mL of a cell culture that had been incubated for 24 h. Cells were heated at 70 °C for 5, 10, 15, or 20 min and were then centrifuged at 3,000 × g for five min. The supernatant was then transferred to a new reagent tube, and 100 μL of the XTT/menadione solution was added to both the cells and the supernatant, followed by incubation in the dark for 2 h at 36 °C and repeated centrifugation. Subsequently, 75 μL of supernatant was transferred to a 96-well microtiter plate, and the OD_492_ was measured. The cell concentration and the relative XTT reduction were determined.

### Antibiofilm activity of antiinfective agents and natural substances

This assay was amplified to investigate the susceptibility of the cells of *E. dermatitidis* biofilm against the antiinfective agents colistin, voriconazole, and micafungin and against the QSM farnesol and the natural compounds 1,2,3,4,6-pentagallolyl glucose (PGG) and epigallocatechin-3-gallate (EGCG). Three *E. dermatitidis* strains with high ability to form biofilm were used. The MIC of voriconazole and the MEC of micafungin against *C. albicans* were determined by E-test. All other MICs against *C. albicans* and *E. dermatitidis* as well as the MEC of micafungin against *E. dermatitidis* were investigated by microdilution method according to EUCAST document for moulds^35^. The test ranges of antifungal agents and natural compounds used in the microdilution assay were as follows: Voriconazole (Sigma, Steinheim, Germany) and micafungin (MedChem Express, New Jersey, USA) 0.0312–16 mg/L, colistin (Sigma, Steinheim, Germany) 1–512 mg/L and farnesol, EGCG and PGG (all from Sigma, Steinheim, Germany) 4–2048 mg/L. *Exophiala dermatitidis* cells were treated with the total, one-half, one-quarter and one-eighth of the concentration of the MIC/MEC. We tested the activity of the six agents in three assay variations against *E. dermatitidis* adhesion to surface, against biofilm development, and against mature biofilm.

The antiinfective and natural agents were diluted in 1% solvent (dimethyl sulfoxide; DMSO) and RPMI medium for the preparation of a stock solution. The working solutions were made by dilution with sterile RPMI and the stock and working solutions were stored at −18 °C until use, with one exception, the solutions containing micafungin were stored at −80 °C. The compounds to be tested were added to the wells of a polystyrene, sterile, flat-bottomed 96-well microtiter plate at amounts of 100 μL.

We investigated the anti-adhesion effect by treating the fungus with drugs from the beginning of biofilm formation. We prepared the microtiter plates with the substances in advance. For the assay, the prepared plates were inoculated with 100 μL of cell suspension prepared fresh as described above, and cell counts in form of CFUs were carried out. After 2 h of incubation at 35 °C, the plates were incubated with XTT and evaluated as described above.

We evaluated the activity of the substances against the development of biofilm, as described above for the anti-adhesion effect, except for a difference in the time point of treatment with the substances. We placed 200 μL cell suspensions in each well and incubated it for 2 h at 35 °C. Subsequently, the plate was rinsed thrice with sterile PBS, and 100 μL of drug/substance solution was added to each well. The plates were incubated for 48 h until the XTT assay was performed as described above.

We tested the activity of the substances against mature biofilms over 48 h at 35 °C. The procedures were the same as those for the biofilm inhibiting effect, but in this case the drugs were added after two days of biofilm formation.

To investigate the *in vitro* synergy of micafungin and colistin, we applied the checkerboard technique to the assay to obtain the fractional biofilm eradication concentration (FBEC)^36^. We serially diluted micafungin along the ordinate while serially diluting colistin along the abscissa. Final drug concentrations were applied as described above. For data analysis, we determined the MBEC at which 50% of the biofilm was reduced (MBEC_50_) for both agents when used alone and in combination. The FBEC was determined using the following formula:


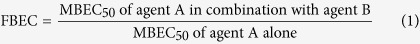


The FBEC index (ΣFBEC) is the sum of the FBEC of agent A and the FBEC of agent B. A ΣFBEC index smaller than or equal to 0.5 indicates synergism; a ΣFBEC index between 0.51 and 0.6 indicates partial synergism; a ΣFBEC index between 0.61 and 4.0 indicates indifference; and a ΣFBEC index higher than 4 indicates antagonism^36^.

### Microscopy images

CLSM was used to visualize the biofilm of *E. dermatitidis.* Biofilms of *E. dermatitidis* were formed over 48 hours at 35 °C in sterile, 1 μ-Slide 4 Well Glass Bottom dishes (ibidi GmbH®, Martinsried, Germany) with an inoculum of 1 × 10^6^ cells/mL in RPMI. The supernatant was discarded and the cells were fixed with 100% methanol for 2 min. The methanol was discarded and the cells were air-dried before the biofilm was stained with 0.01% acridine orange (Becton Dickenson, New Jersey, USA) for 3 min. Subsequently, the biofilm was rinsed three times with PBS and air-dried again. The cells were observed in the ELYRA LSM 710 (Zeiss, Oberkochen, Germany) with a laser at 488 nm and a 20 × objective. The images were processed using the Zen black software (Zeiss, Oberkochen, Germany). Two dimensional and two and a half dimensional (2.5D = pseudo-3D) images were created.

### Statistical analysis

Values are presented as the mean obtained from three separate observations. Values were compared with Student’s *t*-test; statistical significance was set at the level of *P* < 0.05. Statistical analysis was performed with GraphPad Prism 6 (GraphPad Software Inc., La Jolla, CA, USA).

## Additional Information

**How to cite this article:** Kirchhoff, L. *et al*. Biofilm formation of the black yeast-like fungus *Exophiala dermatitidis* and its susceptibility to antiinfective agents. *Sci. Rep.* **7**, 42886; doi: 10.1038/srep42886 (2017).

**Publisher's note:** Springer Nature remains neutral with regard to jurisdictional claims in published maps and institutional affiliations.

## Supplementary Material

Supplementary Information

## Figures and Tables

**Table 1:** Average minimum inhibitory concentration (MIC) values (in mg/L) for voriconazole, 1,2,3,4,6-penta-O-galloyl-β-d-glucose (PGG), epigallocatechin gallate (EGCG), colistin and farnesol and the minimum effective concentration (MEC) of micafungin against three *E. dermatitidis* isolates. (P1, P2, CF2) and *C. albicans* (ATCC 90028) detected by microdilution method after 48 hours of incubation at 35 °C.

	Voriconazole mg/L	PGG mg/L	EGCG mg/L	Farnesol mg/L	Colistin mg/L	Micafungin mg/L
P1	0.25	2048	1024	512	64	8
P2	>16	>2048	>2048	2048	128	8
CF2	>16	>2048	>2048	>2048	>512	8
*C. albicans*	0.023	>2048	>2048	>2048	>512	0.016

**Table 2:** List of included fungal isolates.

Isolate abbreviation	Species	Reference Number #	Isolate origin
E1	*Exophiala dermatitidis*	CBS 120550	Steam bath, Austria
E2	*Exophiala dermatitidis*	CBS 736.87	Beer, Ireland
E3	*Exophiala dermatitidis*	CBS 109142	Berries, Netherlands
E4	*Exophiala dermatitidis*	CBS 109143	Sauna, Netherlands
E5	*Exophiala dermatitidis*	CBS 120479	Air, Germany
E6	*Exophiala dermatitidis*	CBS 106.92	Public pool, Japan
E7	*Exophiala dermatitidis*	CBS 120435	Steam bath, Thailand
E8	*Exophiala dermatitidis*	CBS 120574	Sauna, Thailand
CF 1	*Exophiala dermatitidis*	CBS 154.90	CF, Sputum, Aachen, Germany
CF 2*	*Exophiala dermatitidis*	CBS 552.90	CF, Sputum, Aachen, Germany
CF 3	*Exophiala dermatitidis*	CBS 149.90	CF, Sputum, Aachen, Germany
CF 4	*Exophiala dermatitidis*	CBS 120429	Finland
CF 5	*Exophiala dermatitidis*	Lfd. Nr.1951	CF, Sputum, Essen, Germany
CF 6	*Exophiala dermatitidis*	Lfd. Nr. 1946	CF, Sputum, Essen, Germany
CF 7	*Exophiala dermatitidis*	Lfd. Nr. 1952	CF, Sputum, Essen, Germany
CF 8	*Exophiala dermatitidis*	CBS 120155	CF, Belgium
CF 9	*Exophiala dermatitidis*	CBS 100337	CF, Sweden
CF 10	*Exophiala dermatitidis*	CBS 551.90	CF, Germany
CF 11	*Exophiala dermatitidis*	CBS 550.90	CF, Germany
CF 12	*Exophiala dermatitidis*	CBS 549.90	CF, Germany
CF 13	*Exophiala dermatitidis*	CBS 748.88	CF, Norway
CF 14	*Exophiala dermatitidis*	CBS 148.90	CF, Germany
CF 15	*Exophiala dermatitidis*	CBS 213.90	CF, Germany
CF 16	*Exophiala dermatitidis*	CBS 156.90	CF, Germany
CF 17	*Exophiala dermatitidis*	CBS 153.90	CF, Germany
CF 18	*Exophiala dermatitidis*	Lfd Nr. 1741	CF
CF 19	*Exophiala dermatitidis*	Lfd Nr. 1742	CF
CF 20	*Exophiala dermatitidis*	Lfd Nr. 1802	CF, Sputum
CF 23	*Exophiala dermatitidis*	Lfd Nr. 1873	CF, Sputum
CF 24	*Exophiala dermatitidis*	Lfd Nr. 1874	CF, Sputum
CF 25	*Exophiala dermatitidis*	Lfd Nr. 1875	CF, Sputum
CF 26	*Exophiala dermatitidis*	Lfd Nr. 1883	CF, Sputum
CF 28	*Exophiala dermatitidis*	Lfd Nr. 1889	CF, Sputum
CF 31	*Exophiala dermatitidis*	Lfd Nr. 1909	CF
CF 32	*Exophiala dermatitidis*	Lfd Nr. 1910	CF
CF 33	*Exophiala dermatitidis*	Lfd Nr. 1911	CF
CF 34	*Exophiala dermatitidis*	Lfd Nr. 1912	CF
CF 35	*Exophiala dermatitidis*	Lfd Nr. 1913	CF
CF 36	*Exophiala dermatitidis*	Lfd Nr. 1914	CF
CF 37	*Exophiala dermatitidis*	Lfd Nr. 1915	CF
CF 38	*Exophiala dermatitidis*	Lfd Nr. 1916	CF
CF 39	*Exophiala dermatitidis*	Lfd Nr. 1917	CF
CF 41	*Exophiala dermatitidis*	Lfd Nr. 1919	CF
CF 43	*Exophiala dermatitidis*	Lfd Nr. 1921	CF
P1	*Exophiala dermatitidis*	CBS 109154	Brain, Japan
P2	*Exophiala dermatitidis*	CBS 116372	Brain, Japan
P4	*Exophiala dermatitidis*	CBS 578.76	Chromomykose, Taiwan
P5	*Exophiala dermatitidis*	CBS 577.76	Cervical lymphnodes, Taiwan
P6	*Exophiala dermatitidis*	CBS 579.76	Brain, Japan
P7	*Exophiala dermatitidis*	CBS 207.35	Chromomykose, Japan
P8	*Exophiala dermatitidis*	NCPF* 2542	Human lung
P9	*Exophiala dermatitidis*	CBS 120546	Skin graft, Greece
P10	*Exophiala dermatitidis*	CBS 109148	Human faeces, Netherlands
P11	*Exophiala dermatitidis*	CBS 120473	Brain, USA
P12	*Exophiala dermatitidis*	CBS 100341	Blood, Germany
P13	*Exophiala dermatitidis*	CBS 109153	External ear, Finland
P14	*Exophiala dermatitidis*	CBS 120542	Human faeces, Slovenia
P15	*Exophiala dermatitidis*	CBS 424.67	Chromomycosis, Germany
P16	*Exophiala dermatitidis*	CBS 109153	External ear, Finland
WT	*Exophiala dermatitidis*	ATCC 34100	Wild type of mutants, unknown origin
Mu2	*Exophiala dermatitidis*	ATCC44504	Mel^-^3, derived from ATCC34100
*C. albicans*	*C. albicans*	ATCC 90028	Blood, Iowa, USA

E = environmental isolate. CF = isolate from cystic fibrosis (CF) patients. P = isolate from non-CF patients.

^*^This isolate was excluded in the CV assay.
