# Supplementary material for: Biofilm formation of the black yeast-like fungus Exophiala dermatitidis and its susceptibility to antiinfective agents
Source: Sci Rep. 2017 Feb 17;7:42886. doi: 10.1038/srep42886 (PMC5314418; doi:10.1038/srep42886)
Supplement: Supplementary Information [file srep42886-s1.doc]

**Biofilm formation of the black yeast-like fungus *Exophiala dermatitidis* and its susceptibility to antiinfective agents**

Lisa Kirchhoff^1#^, Maike Olsowski^1#^, Katrin Zilmans^1^, Silke Dittmer^1^, Gerhard Haase^2^, Ludwig Sedlacek^3^, Eike Steinmann^4^, Jan Buer^1^, Peter-Michael Rath^1^ & Joerg Steinmann^1*^

^1^ Institute of Medical Microbiology, University Hospital Essen, University of Duisburg-Essen, Essen, Germany

^2^ Institute of Medical Microbiology, Rheinisch-Westfälische Technische Hochschule Aachen University Hospital, Aachen, Germany

^3^ Institute of Medical Microbiology and Hospital Epidemiology, Medical School Hannover (MHH), Hannover, Germany

^4^ Institute for Experimental Virology, TWINCORE Centre for Experimental and Clinical Infection Research; a joint venture between the Medical School Hannover (MHH) and the Helmholtz Centre for Infection Research (HZI)

*****Corresponding author:

Joerg Steinmann, MD, Institute of Medical Microbiology, University Hospital Essen, University of Duisburg-Essen, Hufelandstr. 55, 45122 Essen, Germany

Telephone: + 49-201-72385771

Fax: + 49-201-7235602

E-Mail: joerg.steinmann@uk-essen.de


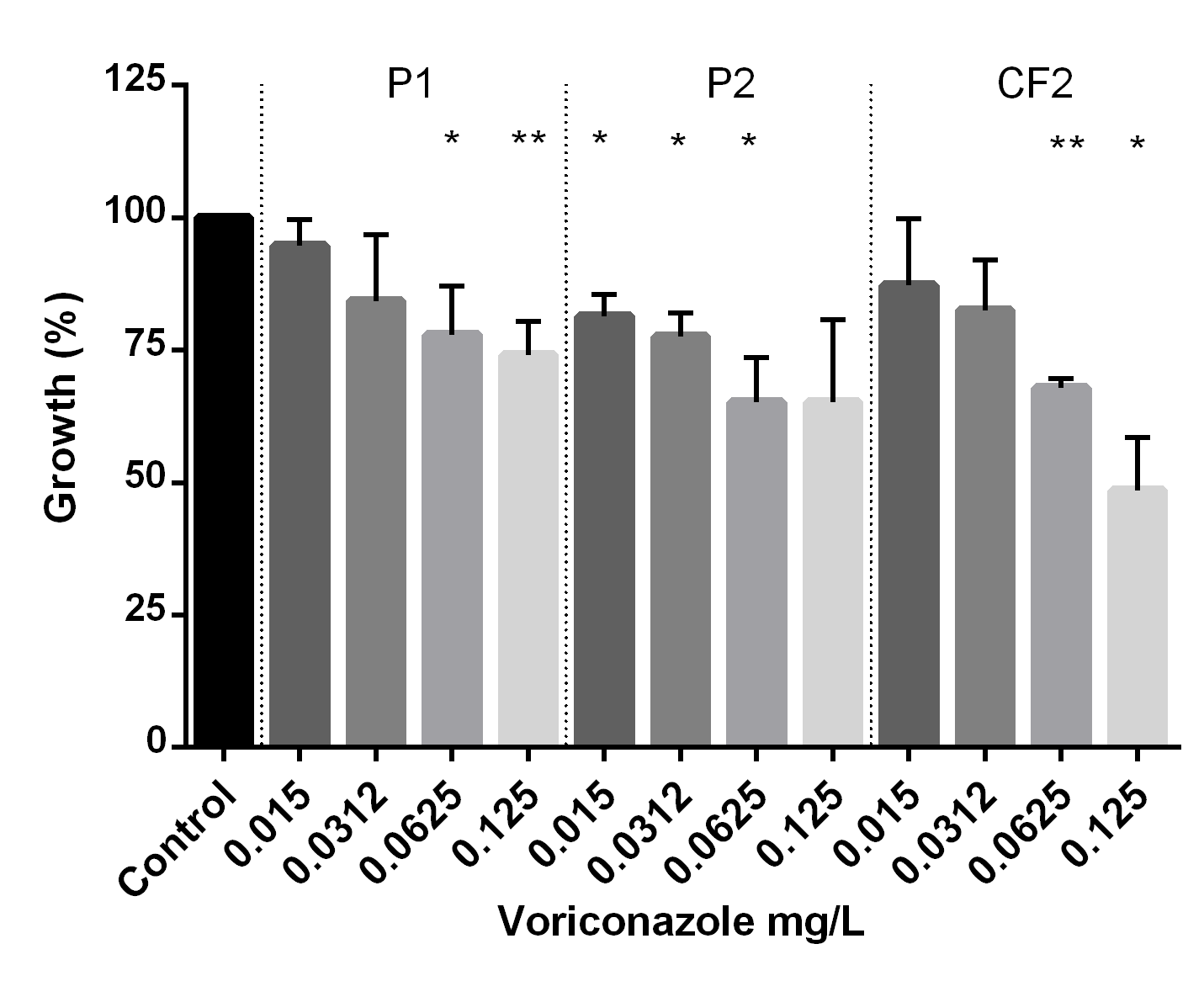


Fig. S 1 Growth (mean with standard deviation in %) of *E. dermatitidis* 48-hour preformed biofilm after treatment with voriconazole for 48 hours at concentrations of 0.125 mg/L, 0.0625 mg/L, 0.0312 mg/L, and 0.0156 mg/L. P1 = *E. dermatitidis* CBS 109154. P2 = *E. dermatitidis* CBS 116372. CF2 = *E. dermatitidis* CBS 552.90. Control = growth control without treatment. Growth was measured by XTT assay; optical density readings at 492 nm (OD_492_) were measured. * *P* < 0.05; ** *P* < 0.05; *** *P* < 0.001. n = 3.

Table S 1: Minimum biofilm eradication concentration and fractional biofilm eradication concentration at which 50% of biofilm is reduced (MBEC_50_ and FBEC_50_), as well as the sum of the FBEC_50_ (∑FBEC_50_), by micafungin and colistin against *E. dermatitidis* cells, treated before adhesion. Analysed Isolates: P1 (CBS 109154), P2 (CBS 116372) CF2 (CBS 552.90). Incubation of cells for adhesion over 2 hours at 35 °C. Concentrations in mg/L. Tested agent concentration ranged from 1 to 8 mg/L micafungin and 8 to 64 mg/L colistin.

|  | **MBEC_50_ alone**  **mg/L** | **MBEC_50_ in combination**  **mg/L** | **FBEC_50_**  **mg/L** | **∑FBEC_50_**  **mg/L** |
| --- | --- | --- | --- | --- |
| **Micafungin** | 1 | 1 | 1 | 1.0156 |
| **Colistin** | >64 | 1 | 0.0156 |

Table S 2: Minimum biofilm eradication concentration and fractional biofilm eradication concentration at which 50% of biofilm is reduced (MBEC_50_ and FBEC_50_), as well as the sum of the FBEC_50_ (∑FBEC_50_), by micafungin and colistin against *E. dermatitidis* biofilm cells, treated after 48 hours of biofilm formation at 35 °C. Analysed Isolates: P1 (CBS 109154), P2 (CBS 116372) CF2 (CBS 552.90). Incubation of cells for 48 hours at 35 °C. Concentrations in mg/L. Tested agent concentration ranged from 1 to 8 mg/L micafungin and 8 to 64 mg/L colistin.

|  | **MBEC_50_ alone**  **mg/L** | **MBEC_50_ in combination**  **mg/L** | **FBEC_50_**  **mg/L** | **∑FBEC_50_**  **mg/L** |
| --- | --- | --- | --- | --- |
| **Micafungin** | 8 | 1 | 0.125 | 1.125 |
| **Colistin** | 64 | 64 | 1 |
